# Relationship between the exposure to cumulative cardiovascular health behaviors and factors and chronic kidney disease—The Kailuan study

**DOI:** 10.1371/journal.pone.0203171

**Published:** 2018-08-31

**Authors:** Jinhong Hou, Junjuan Li, Jinjie Huang, Chunhong Lu, Jing Zhou, Yang Liu, Shouling Wu, Fang Wei, Lihua Wang, Haiyan Chen, Haibo Yu, Zhe Wang, Aili Jiang

**Affiliations:** 1 Kidney disease and blood purification department, The Second Hospital of Tianjin medical university, Tianjin, China; 2 Department of Nephrology, Kailuan General Hospital, Tangshan, China; 3 Department of Cardiology, Kailuan General Hospital, Tangshan, China; Edith Cowan University, AUSTRALIA

## Abstract

It is unclear whether ideal cardiovascular health (CVH) behaviors and factors, particularly cumulative exposure to ideal CVH (cumCVH), is associated with chronic kidney disease (CKD). The aim of the study was to examine the effect of cumCVH on CKD using the data from the Kailuan study. The study included the 27,970 (21,199 males) of the Kailuan community (China). The participants were 19 to 98 years of age. They were followed in 2008–2009, 2010–2011, and 2012–2013 by the same medical staff that did the initial physical examinations in 2006–2007. Participants were censored on the visit reporting CKD. A CVH score was created based on the seven AHA health metrics. The cumCVH score was CVH_1_×time_v1-v2_+CVH_2_×time_v2-v3_+CVH_3_×time_v3-v4_. In the fully adjusted model, compared with the lowest quintile of cumCVH, individuals in the highest quintile had a 75% lower risk of CKD (95% confidence interval (CI): 66–82%). Every additional year lived with a 1-unit increase in ideal CVH was associated with an 11% (95% CI: 9–13%) reduction in the incidence of CKD. Furthermore, when we excluded each of the six metrics from the cumCVH score in turn, the association was unaffected after the exclusion of individual risk factors. Ideal CVH is associated with a reduced incidence of CKD. Measurements of cumCVH are more likely to reflect the lifetime risk of CKD and possibly of other health outcomes.

## Introduction

The worldwide prevalence of chronic kidney disease (CKD) is about 8–16% [1] and up to 10.8% in Chinese adults [2]. Diabetes mellitus [3], hypertension [3–5], hypercholesterolemia [6], and high body mass index [7] are the main causes of death or cardiovascular events, but also of CKD as well as of the prevalence, morbidity, and mortality of cardiovascular complications of CKD [8–13]. Nevertheless, exercising can decrease the development of CKD and the risk of mortality.

In 2010, the American Heart Association defined seven cardiovascular health (CVH) behaviors and factors (smoking status, body mass index, physical activity, healthy dietary score, total cholesterol, blood pressure, and fasting blood glucose) that are considered to improve the cardiovascular death [14,15]. Recent studies showed that the CVH behaviors and factors are relevant to atherosclerosis and cardiovascular events [16,17], but the relationship between CVH and CKD is less well understood. In addition, the occurrence and development of CKD take a long time, and many available data do not adequately assess the sustained effect of long-term cumulative exposure on CKD. The Framingham Heart Study [18] showed that the assessment of the duration and grade of the CVH metrics as measure for the cumulative exposure was more accurate than a cross-sectional analysis in examining the relationship between the CVH metrics and diseases. Meantime, higher CVH cumulative exposure is protective against atherosclerosis, diabetes mellitus, and hypertension [19–22]. Zemaitis et al. [23] showed that among patients with atherosclerosis, higher blood pressure cumulative exposure does harm the kidneys.

Therefore, because of the lack of data regarding the relationship between CVH and CKD, the present study aimed to examine the effect of CVH cumulative exposure on CKD using the data from the Kailuan study (registration: ChiCTR-TNC-11001489), which is a prospective cohort study carried out in the Kailuan community in Tangshan City (China). The 101,510 participants were recruited between June 2006 and October 2007 and were followed every 2 years for three follow-ups (2008–2009, 2010–2011, and 2012–2013) [22].

## Materials and methods

### Subjects

The study included the 101,510 (81,110 males) of the Kailuan community. The participants were 19 to 98 years of age. They were followed in 2008–2009, 2010–2011, and 2012–2013 by the same medical staff that did the initial physical examinations in 2006–2007. The original Kailuan study was a prospective study, but the present study is a secondary analysis with a retrospective design. This study was designed to investigate the effects of cumulative CVH (cumCVH) on CKD. The CVH scores during the first three physical examinations were summed up and their cumulative effects on CKD were analyzed. Patients who did not attend all follow-up examinations were excluded because the cumulative score could not be calculated.

The inclusion criteria were: 1) employees who attended all four physical examinations; and 2) agreed participate in the study and signed the informed consent form. The exclusion criteria were: 1) renal lesions in 2006–2007; 2) missing initial CVH data; 3) loss to follow-up; 4) missing CVH data during follow-up; 5) missing serum creatinine and urine protein data; or 5) CKD diagnosed at the 2^nd^ or 3^rd^ examination.

The original study and subsequent substudies were approved by the ethics committee of Kailuan General Hospital. Each participant provided a written informed consent. The study was conducted according to the guidelines of the Declaration of Helsinki.

### Data collection

Epidemiological, anthropometric, and biochemistry data were collected as previously described [22,24]. Study participants were asked to fast for more than 8 h and blood samples (5 mL) were taken at 7:00–9:00 am the following morning. Blood samples were centrifuged at room temperature for 10 min at 3000 ×g. An enzymatic method (Shanghai Mingdian Bioengineering, Shanghai, China) was used to test serum creatinine using a Hitachi 7600 autoanalyzer (Hitachi, Tokyo, Japan) within 4 h of serum isolation. The intra- and inter-assay coefficients of variation (CV) were <10%. The assay linearity ranged from 44 to 106 μmol/L. The tests were performed by professional laboratory staff in strict accordance with the requirements of the Ministry of Health.

The routine urine test was carried on by urine dry chemistry and urine sediment test using the H12-MA test paper and a DIRUI-600 urine analysis system (Changchun Dirui Medical Co., Ltd., Beijing, China). Proteinuria was tested by a semi quantitative method. Negative means <15 mg/dL; traces mean 15–29 mg/dL; 1+ means 30–300 mg/dL; 2+ means 300–1000 mg/dL; and 3+ means >1000 mg/dL.

### Definitions

Health indicator: The study started in 2006 and there was no data about vegetable consumption in the survey. Meanwhile, considering the effect of salt on the cardiovascular health and the health indicator by Professor Hu, the Kailuan study used salt intake instead of vegetable consumption. The body mass index (BMI) was evaluated according to The Guidance for Prevention and Control for Overweight and Obesity from the Ministry of Health. The optimal amount of exercise was defined as 150 min of moderate intensity exercise every week or 75 min of high intensity exercises every two weeks, or both.

Health behaviors: 1) smoking: i) ideal: never; ii) intermediate: not presently; and iii) poor: currently smoking; 2) BMI: i) ideal: <24 kg/m^2^; ii) intermediate: 24–27.9 kg/m^2^; and iii) poor: ≥28 kg/m^2^; 3) exercise: i) ideal: regular (>3 times every week and >30 min each time); ii) intermediate: irregular (≤3 times every week or <30 min each time); iii) poor: never; and 4) healthy diet: i) ideal: no additional salt; ii) adding salt occasionally; and iii) poor: salty.

Health elements: 1) total cholesterol (TC): i) ideal: TC <5.18 mmol/L without medical treatment; ii) intermediate: TC 5.18–6.21 mmol/L without treatment or <5.18 mmol/L with medical treatment; and iii) poor: TC ≥6.22 mmol/L; 2) blood pressure (BP): i) ideal: systolic BP (SBP) <120 mmHg and diastolic BP (DBP) <80 mmHg without medical treatment; ii) intermediate: SBP 120–139 mmHg and DBP 80–89 mmHg without treatment or BP <140/90 mmHg with medical treatment; and iii) poor: SBP ≥140 mmHg or DBP ≥90 mmHg. and 3) fasting blood glucose (FBG): i) ideal: FBG <6.1 mmol/L without medical treatment; ii) intermediate: FBG 6.1–6.9 mmol/L without treatment or <6.1 mmol/L with medical treatment; and iii) poor: FBG ≥7.0 mmol/L.

The estimated glomerular filtration rate (eGFR) was estimated using the two-race CKD-EPI formula [19]. Females: when serum creatinine (Scr) ≤62 μmol/L, eGFR = 144×(Scr/0.7)-0.329×(0.993)age; when serum creatinine >62 μmol/L, eGFR = 144×(Scr/0.7)-1209×(0.993)age. Males: when serum creatinine ≤80 μmol/L, eGFR = 141×(Scr/0.9)-0.411×(0.993)age; when serum creatinine >80 μmol/L, eGFR = 141×(Scr/0.9)-1.029×(0.993)age.

CKD was defined as eGFR <60 ml/min/1.73 m^2^ and/or positive proteinuria [9–12]. For those who had abnormal physical examination results, we suggested them to visit the relevant clinic of our hospital and affiliated hospitals to confirm the diagnosis and receive the appropriate treatments.

### Health score

To examine the cumulative exposure of the seven CVH metrics, we created a categorical variable for each component of the health metrics: “ideal” was coded as 2, “intermediate” was coded as 1, and “poor” was coded as 0. The total ideal CVH score of each individual was the sum score of the seven ideal CVH metrics and ranged from 0 to 14. The cumCVH score was defined as the summed CVH score for each examination (baseline or follow-up examination) multiplied by the time between the two consecutive visits in years: CVH_1_×time_v1-v2_+CVH_2_×time_v2-v3_+CVH_3_×time_v3-v4_, where CVH_1_, CVH_2_, and CVH_3_ indicate the CVH score at examinations #1 (baseline), #2, and #3, while time_v1-v2_, time_v2-v3_, and time_v3-v4_ indicate the participant-specific time interval between the consecutive examinations #1 to #4, in years. The participants were divided into five categories based on the quintiles of the cumCVH scores. Hence, cumCVH was categorized as <52, 52–58, 59–63, 64–68, and ≥69 points.

### Follow-up

The participants were followed every two years. The participants who developed CKD were censored at the visit reporting CKD. Follow-up was censored on October 31^st^, 2013, i.e. on the fourth physical examination.

### Statistics analysis

Medical data were entered at each evaluation centers and stored in the study database (Oracle 10.2g) hosted on a server at Kailuan Hospital and analyzed using SAS 9.3 (SAS Institute, Cary, NY, USA). Continuous data were tested for normal distribution using the Kolmogorov-Smirnov test. Normally distributed continuous variables were presented using means ± standard deviation (SD) and compared using one-way ANOVA or the rank-sum test, as appropriate. Non-normally distributed continuous variables were presented as median (interquartile range) and analyzed using the Kruskal-Wallis test. Categorical variables were presented as n (%) and compared using the chi-square test. Multivariate logistic regression analysis was used to analyze the factors affecting CKD, and presented as odds ratio (OR) and 95% confidence interval (CI). Two-sided P-values <0.05 were considered statistically significant.

## Results

### Participants

Among the 101,510 individuals who took part in the 2006–2007 physical examinations, 1200 were diagnosed with CKD and 15,589 had missing data about baseline ideal CVH. Excluding them, 84,721 individuals were included in the analysis. Among them, 21,222 did not attend the 2^nd^ physical examination for different reasons, 875 had missing CVH data, 7,960 had missing serum creatinine and proteinuria data, and there were 4245 new CKD patients. Then, 12,140 individuals did not attend the 3^rd^ physical examination for different reasons, 176 had missing CVH data, 2890 had missing serum creatinine and proteinuria data, and there were 1247 new CKD patients. Then, 5996 individuals did not attend the 4^th^ physical examination for different reasons. Finally, 27,970 individuals were included in the analysis (S1 Table). Mean age was 48.0±11.4 years; 75.8% of them were male. The median CVH score at baseline was 9 (7–10) points (S1 Table).

S1 Table shows the comparison of the characteristics of the participants and non-participants. The participants (48.0±11.4 years) were younger than the excluded participants (53.4±12.8 years), had a higher level of education (31.16% vs. 27.67%, P<0.001), and had lower levels of hsCRP (S1 Table).

### cumCVH scores

The participants were categorized into five groups according to the exposure of cumCVH. With the increase of cumCVH, the proportions of female, high education background, high salary, and no alcohol intake became higher and the levels of uric acid and high-sensitive CRP became lower (all P<0.001) (Table 1).

**Table 1:** Characteristic of the participants in 2006 according to the cumulative exposure of CVH.

	Group of cumulative exposure of CVH					P
	Q1	Q2	Q3	Q4	Q5	
N	5594	5594	5594	5594	5594	
Cumulative Cardiovascular Health score	<52	52–58	59–63	64–68	≥69	
Age, years	46.4±9.5	47.2±10.4	48.2±11.4	48.8±12.2	49.4±13.0	<0.0001
Men, n (%)	5281 (94.4)	4903 (87.7)	4477 (80.0)	3728 (66.6)	2810 (50.2)	<0.0001
Women, n (%)	313 (5.6)	691 (12.3)	1117 (20.0)	1866 (33.4)	2784 (49.8)	<0.0001
Education, n (%)						<0.0001
Illiteracy/primary school	28 (0.5)	29 (0.5)	24 (0.4)	27 (0.5)	38 (0.7)	
Middle school	4003 (71.6)	4010 (71.7)	3892 (69.6)	3750 (67.0)	3454 (61.7)	
High school or above	1563 (27.9)	1555 (27.8)	1678 (30.0)	1817 (32.5)	2102 (37.6)	
Income, ¥/month, n (%)						<0.0001
<600	2128 (38.1)	1829 (32.7)	1735 (31.0)	1549 (27.7)	1202 (21.5)	
600–800	2540 (45.4)	2891 (51.7)	2977 (53.3)	3133 (56.0)	3274 (58.5)	
≥800	924 (16.5)	870 (15.6)	879 (15.7)	909 (16.3)	1116 (20.0)	
Alcohol drinking, n (%)						<0.0001
Never	1854 (33.2)	2525 (45.2)	3001 (53.7)	3589 (64.2)	4142 (74.1)	
Past	210 (3.8)	183 (3.3)	195 (3.5)	126 (2.3)	119 (2.1)	
Current, <1 times/d	1617 (28.9)	1554 (27.8)	1409 (25.2)	1196 (21.4)	890 (15.9)	
Current, ≥1 times/d	1910 (34.2)	1328 (23.8)	985 (17.6)	678 (12.1)	441 (7.9)	
Uric acid, μmol/L	316±86	303±82	293±79	279±78	267±76	<0.0001
High sensitive C-reactive protein, mg/L	0.90 (0.40–2.12)	0.72 (0.30–1.80)	0.67 (0.25–1.70)	0.60 (0.20–1.59)	0.46 (0.20–1.25)	<0.0001
Cardiovascular health score	7 (5–8)	8 (7–9)	9 (8–10)	10 (9–11)	11 (10–12)	<0.0001
CKD, n (%)	197 (3.52)	115 (2.06)	128 (2.29)	109 (1.95)	59 (1.05)	<0.0001
eGFR, ml/min/1.73 m^2^	90.5±17.2	89.8±17.2	88.8±17.1	87.8±17.1	86.6±16.5	<0.0001

CVH: ideal cardiovascular health; Q1: quintile 1; Q2: quintile 2; Q3: quintile 3; Q4: quintile 4; Q5: quintile 5; CKD: chronic kidney disease; eGFR: estimated glomerular filtration rate.

### CKD according to cumCVH

The logistic regression analysis showed that with the increase of cumCVH exposure, the frequency of CKD got lower: 3.52%, 2.06%, 2.29%, 1.95%, and 1.05% in increasing quintiles of CVH (P<0.0001) (Table 1). After adjustment for age, gender, education background, salary, alcohol, BVA, and hsCRP levels and compared with the first quintile, the frequency of CKD was still significantly decreasing with cumCVH. The ORs (95% CI) of cumCVH Q2-5 were 0.54 (0.43–0.69), 0.58 (0.46–0.76), 0.47 (0.37–0.61), and 0.25 (0.18–0.34) (Table 2). For each additional 1 point of exposure, the risk of CKD was reduced by 2% (Table 2). Similar results were observed in males and females. If the participants are categorized according to age (<40, 40–59, ≥60), it can be seen that cumCVH played a protective role for CKD. In the 40–59 and ≥60 years groups, the risk of CKD with the highest level of cumCVH was decreased by 83% and 70% respectively, and OR (95% CI) were 0.17 (0.11–0.28), and 0.21 (0.10–0.45) (Table 2).

**Table 2:** Odds ratios and 95% confident intervals of CKD in relation to quintiles of cumulative exposure of CVH.

	Group of cumulative exposure of CVH					One score increase	P for trend
	Q1	Q2	Q3	Q4	Q5		
Total, n	5594	5594	5594	5594	5594		
CKD, n (%)	197 (3.52)	115 (2.06)	128 (2.29)	109 (1.95)	59 (1.05)		
Model 1*	1.00	0.55 (0.43–0.69)	0.58 (0.46–0.73)	0.47 (0.37–0.60)	0.24 (0.18–0.33)	0.98 (0.98–0.99)	<0.0001
Model 2†	1.00	0.54 (0.43–0.69)	0.57 (0.45–0.72)	0.46 (0.36–0.59)	0.24 (0.17–0.32)	0.98 (0.98–0.99)	<0.0001
Model 3‡	1.00	0.54 (0.43–0.69)	0.58 (0.46–0.73)	0.47 (0.37–0.61)	0.25 (0.18–0.34)	0.98 (0.98–0.99)	<0.0001
Sex							
Women	16 (0.29)	19 (0.34)	30 (0.54)	35 (0.63)	29 (0.52)		
Model 3‡	1.00	0.53 (0.26–1.07)	0.60 (0.32–1.13)	0.44 (0.24–0.82)	0.27 (0.14–0.51)	0.84 (0.80–0.87)	<0.0001
Men	181 (3.24)	96 (1.72)	98 (1.75)	74 (1.32)	30 (0.54)		
Model 3‡	1.00	0.54 (0.42–0.69)	0.57 (0.44–0.74)	0.48 (0.36–0.64)	0.23 (0.15–0.34)	0.84 (0.80–0.87)	<0.0001
Age, years							
<40	31 (0.55)	11 (0.20)	16 (0.29)	19 (0.34)	15 (0.27)		
Model 3‡	1.00	0.37 (0.19–0.75)	0.57 (0.30–1.06)	0.62 (0.33–1.15)	0.50 (0.23–1.05)	0.89 (0.87–0.91)	<0.0001
40–59	144 (2.57)	79 (1.41)	72 (1.29)	57 (1.02)	24 (0.43)		
Model 3‡	1.00	0.53 (0.40–0.70)	0.50 (0.38–0.68)	0.41 (0.30–0.58)	0.17 (0.11–0.28)	0.89 (0.87–0.91)	<0.0001
≥60	22 (0.39)	25 (0.45)	40 (0.72)	33 (0.59)	20 (0.36)		
Model 3‡	1.00	0.77 (0.42–1.40)	0.86 (0.50–1.48)	0.56 (0.32–0.97)	0.30 (0.16–0.55)	0.89 (0.87–0.91)	<0.0001

CVH: ideal cardiovascular health; Q1: quintile 1; Q2: quintile 2; Q3: quintile 3; Q4: quintile 4; Q5: quintile 5.

* Adjusted for age (years) and sex.

†Adjusted for as model 1 plus education level (elementary school, middle school, high school or above), income level (≥800, 600–800, and <600 ¥/month) and alcohol consumption (never, past, current, <1 times/d or current, ≥1 times/d).

‡ Adjusted for as model 2 plus high sensitive C-reactive protein and uric acid.

### CKD according to cumCVH, time-weighted exposure

The results using the time-weighted cumulative exposure to CVH (Table 3) were highly comparable to the unweighted model (Table 2). In the fully adjusted model, each additional year lived with a 1-unit increase in ideal CVH was associated with a 18% (95% CI: 0.77–0.87) reduction in new onset CKD (Table 3). Furthermore, when we excluded each of the six metrics from the cumCVH score in turn, the associations were unaffected after exclusion of individual risk factors (Table 4).

**Table 3:** Odds ratios and 95% confident intervals of CKD according to the time weighted cumulative exposure of CVH.

	Group of cumulative exposure of CVH					One score increase	P for trend
	Q1	Q2	Q3	Q4	Q5		
Total, n	5594	5594	5594	5594	5594		
CKD, n (%)	197 (3.52)	137 (2.45)	114 (2.04)	91 (1.63)	69 (1.23)		
Model 1*	1.00	0.66 (0.53–0.83)	0.53 (0.42–0.67)	0.41 (0.32–0.54)	0.33 (0.24–0.44)	0.89 (0.87–0.91)	<0.0001
Model 2†	1.00	0.66 (0.53–0.83)	0.52 (0.41–0.67)	0.41 (0.32–0.53)	0.32 (0.24–0.43)	0.89 (0.87–0.91)	<0.0001
Model 3‡	1.00	0.66 (0.53–0.83)	0.53 (0.42–0.67)	0.42 (0.33–0.55)	0.34 (0.25–0.45)	0.89 (0.87–0.91)	<0.0001
Sex							
Women	16 (0.29)	22 (0.39)	24 (0.43)	32 (0.57)	35 (0.63)		
Model 3‡	1.00	0.73 (0.37–1.44)	0.60 (0.31–1.16)	0.54 (0.29–1.02)	0.39 (0.21–0.75)	0.89 (0.87–0.91)	<0.0001
Men	181 (3.24)	115 (2.06)	90 (1.61)	59 (1.05)	34 (0.61)		
Model 3‡	1.00	0.65 (0.51–0.83)	0.53 (0.41–0.68)	0.39 (0.29–0.53)	0.32 (0.22–0.47)	0.89 (0.87–0.91)	<0.0001
Age, years							
<40	28 (0.50)	12 (0.21)	10 (0.18)	19 (0.34)	23 (0.41)		
Model 3‡	1.00	0.44 (0.22–0.87)	0.38 (0.18–0.79)	0.69 (0.37–1.28)	0.62 (0.31–1.24)	0.89 (0.87–0.91)	<0.0001
40–59	137 (2.45)	95 (1.70)	72 (1.29)	45 (0.80)	27 (0.48)		
Model 3‡	1.00	0.68 (0.52–0.90)	0.53 (0.40–0.72)	0.34 (0.24–0.48)	0.23 (0.15–0.36)	0.89 (0.87–0.91)	<0.0001
≥60	32 (0.57)	30 (0.54)	32 (0.57)	27 (0.48)	19 (0.34)		
Model 3‡	1.00	0.72 (0.43–1.21)	0.57 (0.34–0.95)	0.45 (0.27–0.78)	0.37 (0.20–0.67)	0.89 (0.87–0.91)	<0.0001

CVH: ideal cardiovascular health; Q1: quintile 1; Q2: quintile 2; Q3: quintile 3; Q4: quintile 4; Q5: quintile 5; time weighted cumulative exposure of CVH: (CVH_1_×time_1-2_ + CVH_2_×time _2–3_ + CVH_3_×time _3–4_)/ (time_1-2_ + time _2–3_+ time _3–4_).

* Adjusted for age (years) and sex.

†Adjusted for as model 1 plus education level (elementary school, middle school, high school or above), income level (≥800, 600–800, and <600 ¥/month) and alcohol consumption (never, past, current, <1 times/d or current, ≥1 times/d).

‡ Adjusted for as model 2 plus high sensitive C-reactive protein and uric acid.

**Table 4:** Odds ratios (ORs) and 95% confident intervals (95%CI) of CKD according to the cumulative exposure of CVH from 2006 to 2010, after one individual cardiovascular health is removed from the total score.

Removed component	All		Women		Men	
	OR (95%CI) for one score increase	P for trend	OR (95%CI) for one score increase	P for trend	OR (95%CI) for one score increase	P for trend
Smoking	0.979 (0.975–0.983)	<0.0001	0.979 (0.975–0.983)	<0.0001	0.979 (0.975–0.983)	<0.0001
Salt	0.980 (0.976–0.983)	<0.0001	0.980 (0.976–0.984)	<0.0001	0.980 (0.976–0.984)	<0.0001
Physical exercise	0.979 (0.975–0.983)	<0.0001	0.979 (0.976–0.983)	<0.0001	0.979 (0.976–0.983)	<0.0001
Total cholesterol	0.980 (0.976–0.984)	<0.0001	0.980 (0.976–0.984)	<0.0001	0.980 (0.976–0.984)	<0.0001
Blood pressure	0.984 (0.980–0.988)	<0.0001	0.984 (0.980–0.988)	<0.0001	0.984 (0.980–0.988)	<0.0001
Fasting blood glucose	0.983 (0.979–0.987)	<0.0001	0.983 (0.979–0.987)	<0.0001	0.983 (0.979–0.987)	<0.0001
Body mass index	0.981 (0.977–0.985)	<0.0001	0.982 (0.978–0.985)	<0.0001	0.982 (0.978–0.985)	<0.0001

## Discussion

It is unclear whether ideal CVH behaviors and factors, particularly cumCVH, is associated with CKD. Therefore, this study aimed to examine the effect of cumCVH on CKD development using the data from the Kailuan study [20,22]. The results strongly suggest that ideal CVH is associated with a reduced incidence of CKD. Measures of cumulative exposure to ideal CVH are more likely to reflect the lifetime risk of CKD and possibly of other health outcomes.

In recent years, the prevalence of CKD reached 10.8%, similar to the prevalence of diabetes mellitus [25]. High BP and diabetes mellitus are causes of CKD, and have a time-dependent synergistic effect [3–5]. Their co-existence for a long time will have serious effects on the kidneys. Previous studies showed that high exposure level of cumCVH is protective against high BP and diabetes mellitus [20–22]. For each point of cumCVH, the prevalence of high BP decreases by 2% and the prevalence of diabetes mellitus decreases by 24%. To add to these previous results, the present study showed that for each point of cumCVH, the prevalence of CKD decreased by 2% and the prevalence of CKD in the highest quintile of cumCVH was lower by 75% compared with the lowest quintile.

After stratification of the participants according to age and gender, higher exposure score of cumCVH was associated with lower prevalence of CKD in males. One of the most important reasons is probably that the ideal CVH actions and factors are less popular among male than among female, especially smoking. Accordingly, the prevalence of CKD was 2.1% lower if there was no cumulative exposure of smoking, lower than when excluding any other element from the models. Therefore, small changes in cumCVH score will result in great changes in the prevalence of CKD, and stressing the importance of stopping tobacco could be particularly significant in males.

After stratification of the participants according to age, higher exposure score of cumCVH was associated with lower prevalence of CKD in participants <40 years of age. This association could be confounded by the fact that the proportions of older participants were higher in the subgroups of lower salary and lower education; nevertheless, the associations remained significant after adjustment for these factors.

In addition, the results were consistent even when removing any one of the elements in the cumulative exposures. Therefore, the mid- and long-term CVH exposure is not only relevant to CKD, but also can more accurate reflect the long-term effect, so the people should be intervened to prevent CKD as early as possible. As shown in previous studies [26,27], after 1 year of medical intervention, the glycosylated hemoglobin levels of patients with diabetes mellitus decreased from 6.65% to 6.34%, leading to lower proteinuria, BP, and prevalence of the metabolic syndrome, and improved eGFR. A previous Chinese study showed that if the levels of FBG, TC, SBP, and BMI of the lowest cumCVH subgroup could be lowered by 1.1 mmol/l, 1.0 mmol/L, 10 mmHg, and 2.7 kg/m^2^, respectively, these patients could achieve outcomes similar to those of the moderate cumCVH groups. Therefore, for the prevention of CKD, stopping tobacco, doing more exercise, and improving diet should be emphasized [28]. Just like the HeartRescue Program in China [29], interventions should be designed to improve the cardiac prevention of patient with CKD and to achieve a better care of these patients once they develop a cardiac event. The HeartRescue Program in China proved that such interventions are possible in China [29].

The strength of the present study is that it was a large-scale cohort study, but there are still some limitations. First, there was no detailed diet questionnaire. The salt intake was analyzed by a survey filled by the participants themselves, and 24-h urine sodium excretion was not measured; therefore, the results of salt consumption could lack precision. Nevertheless, urine sodium excretion data were available for 1000 participants, and the correlation between urine sodium and self-reported salt consumption was good (r = 0.78). Secondly, the study did not exclude the patients with cardiovascular diseases. A number of cardiovascular drugs may affect BP, blood lipids, and blood glucose. Finally, as a large-scale cohort study, this study excluded a large number of patients, i.e. those who died for various reasons during the follow-up period, those who were diagnosed with CKD during the second or third visit, those lacking data preventing the calculation of the cumCVH. The exclusion of those participants may lead to unstable results. Nevertheless, the conditions were compared between those who finally participated in the statistical analysis and those who did not. We found that those who did not participate in the analysis were older, but there were no significant differences in the cumCVH scores. Therefore, the incidence of CKD may be underestimated to some extent, and the effect of the cumCVH scores on CKD may be underestimated.

## Conclusions

Ideal CVH is associated with a reduced incidence of CKD. Measures of cumulative exposure to ideal CVH are more likely to reflect the lifetime risk of CKD and possibly of other health outcomes.

## Supporting information

S1 Table Clinical characteristics according to the participants who participated or not. (DOCX)
