# Supplementary material for: Relationship between the exposure to cumulative cardiovascular health behaviors and factors and chronic kidney disease—The Kailuan study
Source: PLoS One. 2018 Aug 31;13(8):e0203171. doi: 10.1371/journal.pone.0203171 (PMC6118362; doi:10.1371/journal.pone.0203171)
Supplement: S1 Table — (DOCX) [file pone.0203171.s001.docx]

**S1 Table.** Clinical characteristics according to the participants who participated or not

|  | Participants | Non-participants | P |
| --- | --- | --- | --- |
| N | 27,970 | 73,540 |  |
| Age, years | 48.0±11.4 | 53.4±12.8 |  |
| Male, n (%) | 21,199 (75.8) | 59,911 (81.5) | <0.0001 |
| Female, n (%) | 6771 (24.2) | 13,629 (18.5) | <0.0001 |
| Education, n (%) |  |  | <0.0001 |
| Illiteracy/primary school | 146 (0.52) | 5073 (6.90) |  |
| Middle school | 19,109 (68.32) | 48,121 (65.44) |  |
| High school or above | 8715 (31.16) | 20,346 (27.67) |  |
| Income, ¥/month, n (%) |  |  | <0.0001 |
| <600 | 8443 (30.20) | 19,798 (28.49) |  |
| 600-800 | 14,815 (52.99) | 40,385 (58.11) |  |
| ≥800 | 4698 (16.80) | 9317 (13.41) |  |
| Alcohol drinking, n (%) |  |  | <0.0001 |
| Never | 15,111 (54.06) | 42,596 (60.53) |  |
| Past | 833 (2.98) | 3133 (4.45) |  |
| Current, <1 times/d | 6666 (23.85) | 12,391 (17.61) |  |
| Current, >1 times/d | 5342 (19.11) | 12,253 (17.41) |  |
| Uric acid, µmol/L | 292±82 | 290±85 | 0.0103 |
| High sensitive C-reactive protein, mg/L | 0.66 (0.26-1.70) | 0.90 (0.32-2.50) | <0.0001 |
| Cardiovascular health scores | 9 (7 - 10) | 9 (7 - 10) | <0.0001 |
